# Levothyroxine and lung cancer in females: the importance of oxidative stress

**DOI:** 10.1186/1477-7827-11-75

**Published:** 2013-08-08

**Authors:** Umberto Cornelli, Gianni Belcaro, Martino Recchia, Annarosa Finco

**Affiliations:** 1 Loyola University School of Medicine, Chicago, USA; 2 University of Chieti, Chieti, Italy; 3 University of Lugano, Lugano, Switzerland; 4 Cor Con International-Ox Res Dept, Parma(PR), Italy

**Keywords:** Levothyroxine, Smoking, Oxidative stress, Lung cancer, Dysthyroidism

## Abstract

**Background:**

Levothyroxine (LT_4_) treatment can lead to iatrogenic hyperthyroidism and oxidative stress that can cause patient discomfort. Oxidative stress is also recognized as one of the causes of chronic diseases and cancer.

**Methods:**

The prevalence of breast, colorectal, gastric and lung cancer in 18 Italian Regions during 2010 was correlated with the sales of LT_4_ in 2009. The cancer prevalence was analyzed in women aged 30–84. This age range corresponds to more than 80% of the consumers of the drug and to about 99% of all malignant cancers. The correlation between sales of LT_4_ and cancers was determined with the technique of Density Ellipses. The age and smoking contribution for lung cancer was determined with the Sequential test.

**Results:**

No significant correlation was seen between LT_4_ sales and breast, colorectal and gastric cancers. A significant correlation was instead found for lung cancer (p < 0.05) corrected for smoking and age.

**Conclusions:**

LT_4_ consumption in Italy is about 0.7 boxes/women/year. There is a correlation between lung cancer and LT_4_ treatment and oxidative stress caused by LT_4_ supplementation can be one of the causes. Although we cannot exclude that dysthyroidism needing LT_4_ supplementation might be the ground for lung cancer itself and measuring oxidative stress could be helpful in avoiding excessive use of the drug.

## Background

During studies aimed at assessing the relationship between food intake and metabolic syndrome in Italy, a large number of subjects was found to use levothyroxine (LT_4_) for hypothyroidism.

A survey conducted in USA on the intake of thyroid supplements [1] found that side effects may occur in as many as 20% of treated cases, the most common being palpitation, sweating, agitation/anxiety and daily discomfort which are typical symptoms of iatrogenic hy-perthyroidism.

A clinical study indicated that these side effects were correlated with the increase of plasma hydroperoxides which are markers of oxidative stress [2], and the use of a pool of physiological modulators (PMs) with antioxidant activity was found effective in reducing all these side effects [3].

These findings suggest that the chronic use of LT_4_ is consistent with an overproduction of reactive oxygen species (ROS) caused by the hypermetabolic status provoked by administration of this hormone [4-6].

Achievement of euthyroid status is monitored by measuring TSH, T_3_ and T_4_ plasma levels, mainly in the morning on a empty stomach, a few times in a year, but measuring oxidative stress was never considered a useful tool for adjusting LT_4_ doses. The LT_4_ administration leads to T_4_ and T_3_ peak between one and two hours after the drug is taken [7,8]. These peaks triggers an increase of plasma hydroperoxides which is reduced by the administration of physiological modulators with antioxidant activity [2,3].

The aim of the present research was to correlate the LT_4_ as a chronic oxidative stress generator with the prevalence of four different types of tumors: breast, colorectal, gastric and lung in 18 Italian Regions. Since more than 80% of supplement prescriptions of the supplement are made out to women, the correlation between LT_4_ sales and cancers was calculated for females only.

## Methods

Data on LT_4_ sales, sales in pharmacy in 18 Italian regions in 2009 and 2010, were provided by IMS (Intercontinental Marketing Service). These data were correlated with the prevalence of breast, colorectal, gastric and lung cancers in females in the same regions.

The IMS sex distribution and age group data show that 83.5% of the LT_4_ prescriptions were made out to females, and 94.4% were to patients aged more than 30 years. Public data on cancer incidence, prevalence and mortality in Italy can be obtained on line and are reported by type of cancer, sex/age/Region (http://www.tumori.net). The raw prevalence data (number of cases/100,000 inhabitants) in the of 30–84 years age group was taken and correlated with the drug sales, regardless of the different doses used.

Smoking prevalence was obtained by Istat.it (http://www.istat.it) from and the relative sex distribution from OSSFAD (Osservatorio Fumo Alcol e Droga Istituto Superiore di Sanità- report May 31, 2010). The average prevalence between 2007 to 2009 in the 18 Italian Regions was taken for the evaluation because data before 2007 were not available for all Regions. Aging index was taken by Istat.it.

### Statistical methods

The continuous variables “prevalence” and “sales LT_4_” were analyzed by correlation analysis with the technique of Density Ellipse [9]. The correlations analysis yields only one number, an index designed to give an immediate picture of how closely two variables move together, while the ellipse represents all combinations of X and Y with the same probability density. It is called an isoprobability curve. The straight line represents the axis common to all these level curves. If the bivariate normal distribution concentrates about this major axis, ρ has a higher numerical value. These ellipses are both density contours and confidence curves. As confidence curves, they show where a given percentage of the data is expected to lie, assuming the bivariate normal distribution. The densities of the ellipse were drawn with p = 0.90.

In case of correlation between LT_4_ and cancer the Multiple Regression Analysis was applied followed by the Sequential test (or type ISS) to highlight the contribution of single variables (aging, smoke and LT_4_ treatment).

The statistical analysis applied to data is a multiple regression model with the stepwise specification. The Stepwise feature computes estimates that are the same as those of other least squares platforms, but it facilitates searching and selecting among many models. Sequential Tests show the reduction in residual sum of squares as each effect is entered into the fit. The sequential tests are also called Type I sums of squares (Type I SS). A desirable property of the Type I SS is that they are independent and sum to the regression SS.

## Results

The Italian females population aged between 30 and 84 years was estimated to be about 19.89 million in 2010 (http://dati.istat.it/) and the relevant sales of LT_4_ in 2009 amounts to 13.93 million boxes (total sales 16.68 million boxes without considering the different dosages) corresponding to a yearly consumption of 0.7 boxes/woman.

The data concerning the different cancers, smoking and aging index are reported in Table 1.

**Table 1:** Levothyroxine sales (number of boxes sold), raw prevalence of some cancer in females in 18 different Italian regions in 2010 in relation to the sales of LT_4_ in 2009; average smoking prevalence in women between 2007 and 2009

	**Type of cancer and prevalence (10**^**5**^**) in females**						
**Area**^**a**^	**Sales LT**_**4**_^**b**^	**Breast**	**Colorectal**	**Gastric**	**Lung**	**Smoking**^**c**^	**Aging index**^**d**^
1	1072879	3165.62	776.88	120.04	97.47	21.6	139.9
2	1028179	3118.67	646.35	96.03	68.08	23.0	150.0
3	267126	2669.96	714.53	199.34	100.45	26.5	180.5
4	273291	3210.26	750.28	131.17	72.57	20.0	116.5
5	1438397	1589.44	370.62	76.35	82.94	23.0	120.2
6	1179775	2674.95	625.72	182.42	91.74	26.0	184.1
7	487525	2250.40	416.66	55.21	62.85	20.0	154.8
8	1087091	1418.33	374.09	87.74	60.79	22.0	122.1
9	405995	1579.13	389.64	103.25	54.03	21.0	168,9
10	408040	2722.42	716.09	198.61	84.07	23.0	168.7
11	1879231	2909.03	733.82	174.65	77.41	29.0	141.9
12	467753	2824.49	751.36	90.85	82.19	23.0	234.6
13	2423825	2882.24	557.33	109.31	138.66	29.0	141.6
14	287660	3424.94	837.82	162.56	116.44	23.1	187.4
15	1474482	3269.15	815.26	169.85	94.09	26.0	170.0
16	631585	1442.77	329.72	97.17	44.45	22.8	130.2
17	1682805	1622.33	378.19	99.62	96.59	27.0	96.5
18	183079	1474.73	430.39	100.85	45.98	16.0	148.2

^a^1-Veneto; 2-Piemonte-Valle d’Aosta; 3-Umbria; 4-Trentino Alto Adige; 5-Sicilia; 6-Toscana; 7-Sardegna; 8-Puglia; 9-Abruzzo-Molise; 10-Marche; 11-Lombardia; 12-Liguria; 13-Lazio; 14-Friuli Venezia Giulia; 15-Emilia Romagna; 16-Calabria; 17-Campania; 18-Basilicata;

^b^Sales of LT_4_, boxes regardless to different doses in 2009, data from IMSHealth–ITSSMS–Anno 2009;

^c^average smoking prevalence from 2007 and 2009 (× 100 people);

^d^Aging index in 2010.

The correlation between smoking and the four cancers (data not reported) was statistically significant for lung cancer only (p < 0.05) while for all the other type of cancer was practically inconsistent.

The aging index was shown to be significant for colorectal cancer only (p < 0.05) and not for all the other type of cancer (data not reported).

**Figure 1:**
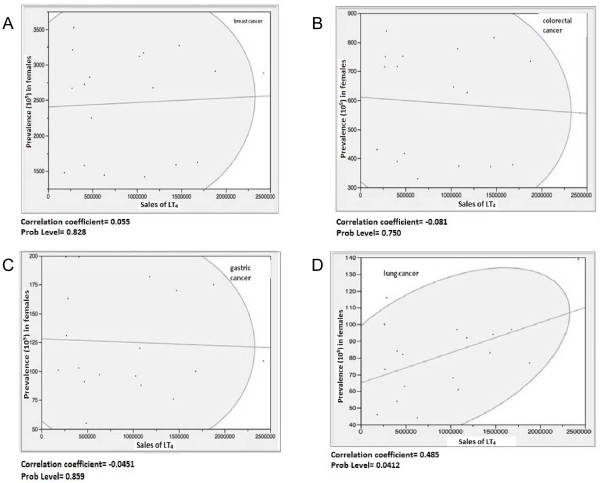
**Levothyroxine sales (number of boxes sold). A)** Raw breast cancer prevalence (10^5^) in female in 18 different Italian Regions. Each point corresponds to a region. **B)** Raw colorectal cancer prevalence (10^5^) in female in 18 different Italian regions. Each point corresponds to a region. **C)** Raw gastric cancer prevalence (10^5^) in female in 18 different Italian regions. Each point corresponds to a region. **D)** Raw lung cancer prevalence (10^5^) in female in 18 different Italian regions. Each point corresponds to a region.

For what concern the correlation between sales of LT_4_ and cancers the data are reported in Figure 1 (A,B,C,D).

The prevalence sales ellipsis is relatively wide, which corresponds to no significant correlation.

The data concerning colorectal cancer and LT_4_ sales (Figure 1B) show that, for this type of tumor, there was no significant relationship and, like in the case of breast cancer, data were spread out over a large ellipsis.

The values of the relationship between sales and prevalence are also distributed over a wide area in the case of stomach cancer (Figure 1C).

A significant (p < 0.05) correlation was found for lung cancer (Figure 1D).

Smoking is known to be related to lung cancer and to cause an over-fitting effect that hides the possible contribution of other variables such as aging and LT_4._ The sequential analysis applied to the data eliminated the effect of smoking and made evident the contribution of aging and LT_4_.

The results were that age do not contribute significantly to the development of lung cancer (p = 0.532) whereas LT_4_ is significantly related to lung cancer (p = 0.003).

A more evident differentiation can be drawn splitting smoking prevalence into three different age range 25–44, 45–64, and 65–84 that was respectively 24.3, 25.9 and 7.4 (OSSFAD 2010 report May 31, 2010). This means that the smoking prevalence was reduced by three times in the last age range. The prevalence of LT_4_ use instead was respectively 18.6, 35.1 and 35.2, showing that in the two age ranges the drug consumption was identical whereas the lung cancer prevalence was respectively 8.1, 64.3 and 199.2. In other words, in the last range, to a drastic reduction of smoking (−71%) was corresponding a sharp increase of lung cancer (+300%).

Sales in 2010 were also compared with the prevalence of cancers in 2010 (instead of sales in 2009-data not reported), and the values were almost identical.

## Discussion

The findings of this research suffer of many limitations due to type of data that analyzed.

For instance, the impossibility to determine the LT_4_ dosage does not allow dose/effect relationship to be looked into. In addition, the lack of sex distribution of the drug in the different regions, the nonexistence of a study protocol and a clinical record form to guide the study may limit further the data interpretation. However, 13.9 million boxes spread between 14 million women are very sizeable figures and may give important indications.

In this assessment no relationship was found between LT_4_ prescription and breast cancer among the Italian population.

The average prevalence measured in the regions of Southern of Italy (Sicilia, Puglia, Abruzzo/Molise, Calabria, Campania, Basilicata, Sardegna) was 1600.65 compared to value of 2998.59 in all the other regions. This difference could be due to Mediterranean diet, which is more widespread in Southern Italy and improves the AO capacity.

The relationship between oxidative stress and this type of cancer was examined in the Long Island Breast Cancer Study Project through the measurement of urinary isoprostane (15-isoprostane F_2t_) which mirrors oxidative stress due to lipid peroxidation [10]. A positive correlation was found between urinary isoprostrane levels and cancer, which underlines the importance of oxidative stress measurement.

It may be possible that the AO intake in the Southern regions of Italy have diluted the pro-oxidant effect of LT_4_ in all the country and made inconsistent the correlation between the two variables under study.

No correlation was found in our study between colorectal cancer and LT_4_ administration. A negative correlation with long term use of LT_4_ (< 5 years) was described in literature in a case–control study on colorectal cancer conducted in Northern Israel [11]; in the case of women, the effect was independent from the use hormone replacement therapy (HRT).

The prevalence of colorectal cancer in the regions of the Southern Italy was lower than in all the other Italy (376.43 and 704.92 respectively), and seems again to be consistent with the fact that the Mediterranean diet is more common in the South of Italy than in the other Italian regions.

Present data do not support any relationship between LT_4_ and gastric cancer. In this case also the regions of Southern of Italy showed a much lower prevalence than all the other Regions (86.30 and 154.93 respectively), suggesting that the Mediterranean diet might be considered a protective tool against this cancer too.

In the Seven Country Study [12] a relationship was found between diet and stomach cancer in two Italian rural population groups. Unfortunately the study was aimed at man only, though the increase in death for gastric cancer was significantly related to high polyunsaturated fatty acid (PUFA) intake that increase oxidative stress [13].

Lung cancer was the only tumor found directly correlated with LT_4_ supplementation. The prevalence in Southern Italy and the rest of the country were 73.53 and 92.89 respectively, with a far lower difference than for the other three cancers.

The importance of smoking, aging and LT_4_ were considered in the multivariate analysis followed by the Sequential test and showed that smoking and LT_4_ were much more responsible for lung cancer than aging. When smoking prevalence was eliminated by the evaluation still the relationship between LT_4_ and lung cancer remained significant; when similar evaluation was conducted with other type of cancer, the correlation was not significant (data not reported).

We may not exclude that the condition of hypothyroidism could favor the development of lung cancer. On the opposite it has been described recently that hypothyroidism reduces the aggressiveness of some cancers because of the presence of thyroid hormone receptors on cancer cells, and spontaneous hypothyroidism may delay onset or reduce aggressiveness of cancers [14]. Recently LT_4_ has been reported as one of the several endogenous factors capable of supporting proliferation of lung cancer cells [15]. The observation that patients with small cell carcinoma of the lung often present symptoms suggestive of hyperthyroidism (i.e. weight loss, anorexia) was made many years ago together with an over production of both T_4_ and T_3_[16]. An old clinical observation on the relationship between lung cancer and thyroid function [17] reported that patients were characterized by a low concentration of T_3_ and an increased T_4_/T_3_ ratio due to a decrease of 5’-monodeiodination (DI). More recently DI activity in lung cancer was found to be lower than in peripheral lung tissue [18].

In experimental animals has been already shown that LT_4_ increases the oxidative stress [19] and spontaneous pulmonary metastases in mice [20]. Furthermore, in rats lung the deiodination of LT_4_ is the lowest compared to all the other tissues [21]. This means that the amount of LT_4_ reaching the lungs following an external supplementation cannot to be transformed into LT_3_ as in the other tissues, and make lungs very vulnerable to possible toxic effects of LT_4._

During the therapy with LT_4_ even at the steady state condition a peak of the hormone is evident a couple of hours after the administration and may cause a temporary condition of hyperthyroidism and a further increase of oxidative stress.

Oxidative stress is well documented in hypothyroidism [22-24] and is even worsened through treatment with LT_4_[1,2]. The difference between the two conditions is that, in case of hypothyroidisms, oxidative stress is due to the reduction of AO [4], whereas, after the LT_4_ treatment it stems from overproduction of ROS from mitochondria [5,6].

A very simple method that can be used to measure oxidative stress is related to hydroperoxides content in plasma which is considered a very reliable test compared to other common methods since it shows very limited coefficient of variation [25].

An inverse association with fruit and vegetables consumption and lung cancer recently has been documented recently in the EPIC study for 50 to 59 age group, without an effect on specific histological subtypes [26].

These data support the importance of the oxidative stress control in lung cancer, as was documented by the increase in urinary isoprostanes in subjects at risk of cancer in the Multiethnic Cohort Study [27].

LT_4_ can alter the oxidative balance in lungs and behave as a negative factor because of oxidative stress, and the condition of oxidative stress should be controlled as a routine measurement.

There should be a reason why oxidative stress taking place during the treatment with LT_4_ seems particularly related to lung cancer only. The hypothesis could be that in lungs the increase of hypoxia-induced factor (HIF-1) which is determined by T_4_ can make oxygen much more available, increasing locally the oxidative stress together with a dangerous angiogenesis stimulation [28-30].

Although we should never forget that LT_4_ is a life-saving thyroid hormone replacement, and that one should not exclude that the pathological reason that leads to the prescription of LT_4_ could favor the lung cancer development also.

However, the impression we get from our experience in epidemiological studies monitoring [31] is that this drug should be prescribed more cautiously. Considering that the population in Italy is about 60 million people and the sales of LT_4_ in the country in 2010 were 17.69 million boxes (+ 6% Vs 2009), hypothyroidism, which is the main reason for the prescription, should be a real national concern (almost 0.7 boxes/women/year).

Most of the time, doctors tell patients treated with LT_4_ that any side effects will be temporary and almost ineluctable, and are usually dealt with through dose reduction. Assessment of oxidative stress and its balance has not been taken into consideration up to now as it should be.

Furthermore in case of oxidative stress and side effects could be important to use different type of thyroid supplementation, such as: liothyronine as well as Armour Thyroid and control the hydroperoxides levels to determine if these compounds are safer than levothyroxine.

A last comment should be made regarding the data stemming from drug sales, which are commonly used to check sales force performance and decide on marketing and sales development strategies. These data can provide extremely helpful information about the risk/benefit ratio of any drug therapy.

## Conclusions

Despite the common knowledge that LT_4_ is a life-saving drug, some concern has to be addressed for its prescription. The findings based on the sales of LT_4_ in Italy are consistent with the hypothesis that there is a direct correlation between this drug and lung cancer, whereas there is no correlation with breast, colorectal and gastric cancer. However, the clinical background of hypothyroidism, which is the main reason for prescribing the drug, might also be involved in the development of lung cancer. Specific epidemiological studies should be conducted to test these two hypothesis.

This work is focused on Italian population, so it should be interesting to conduct similar studies in other regions of the planet and perhaps on patients of different races and subjected to different diets in order to verify if this correlation exist.

## Competing interests

The authors declare that they have no competing interests.

## Authors’ contributions

All authors contributed to the research. All authors read and approved the final manuscript.
